# The Contribution of Oxidative Stress and Inflamm-Aging in Human and Equine Asthma

**DOI:** 10.3390/ijms18122612

**Published:** 2017-12-05

**Authors:** Michela Bullone, Jean-Pierre Lavoie

**Affiliations:** 1 Department of Clinical and Biological Sciences, University of Turin, AUO San Luigi Gonzaga, Regione Gonzole 10, 10043 Orbassano, Italy; michelabullone@gmail.com; 2 Department of Clinical Sciences, Faculty of Veterinary Medicine, Université de Montréal, 3200 Rue Sicotte, St-Hyacinthe, QC J2S 2M2, Canada

**Keywords:** asthma, elderly, neutrophil, oxidative stress, animal model, horse, aging

## Abstract

Aging is associated with a dysregulation of the immune system, leading to a general pro-inflammatory state of the organism, a process that has been named inflamm-aging. Oxidative stress has an important role in aging and in the regulation of immune responses, probably playing a role in the development of age-related diseases. The respiratory system function physiologically declines with the advancement of age. In elderly asthmatic patients, this may contribute to disease expression. In this review, we will focus on age-related changes affecting the immune system and in respiratory structure and function that could contribute to asthma occurrence, and/or clinical presentation in the elderly. Also, naturally occurring equine asthma will be discussed as a possible model for studying the importance of oxidative stress and immun-aging/inflamm-aging in humans.

## 1. Introduction

Globally, the human population is getting older [1,2]. The aging process induces alterations in the structure and the function of several body systems, including the immune system. These alterations affect both the acquired and the innate immunity, dysregulating their response toward exogenous and endogenous stimuli. This is collectively referred to as immunosenescence, or immun-aging [3], and it contributes to the increased susceptibility of the elderly to infections and autoimmune diseases, as well as their blunted response to vaccines [4]. The elderly are also characterized by a subclinical systemic pro-inflammatory state that results from the chronic activation of immune cells in response to the continued antigenic load. This physiological condition is called inflamm-aging [5], and it may predispose to the development of inflammatory diseases [4,6,7,8]. The recent literature suggests that inflamm-aging causes immunosenescence [9]. The efficacy of oxidative metabolism, that is, the ability of endogenous antioxidants to counterbalance the production of reactive oxygen species (ROS) that are physiologically produced by many cellular metabolic processes, also decreases with age, which contributes to establishment of the aging-associated inflammatory milieu (oxi-inflamm-aging) [10,11]. In an increasing number of disorders, a specific aging-associated phenotype is described that has a different clinical profile in the elderly when compared to the younger adult population. Collectively, aging-associated pathologies account for most of the health costs in industrialized countries as they remain poorly characterized, underdiagnosed, and mistreated [2].

Asthma, in the elderly, or geriatric asthma [12], is one of such age-related diseases whose pathophysiology is debated. It is recognized that aging-associated altered immune responses could facilitate the pathogenesis of asthma in this age group [13]. A putative role of oxi-immune-aging in the development of this condition has been hypothesized [14]. Among the body systems, the respiratory apparatus is one of the most exposed to continuous external antigenic load and atmospheric oxygen, which represent risk factors for the development of exaggerated immune responses and oxidative stress manifesting as allergic or other inflammatory reactions, as it occurs in asthma [15]. Given the predominance of macrophages among the inflammatory cell types within the alveoli, and the central role of the macrophages in the phenomenon of inflamm-aging, the lung could be considered as an organ that is at high risk of developing exaggerated inflammatory responses during aging [9]. Asthma in the elderly is a phenotype that is characterized epidemiologically by higher morbidity and mortality rates when compared to adult asthma, and clinically by a neutrophilic rather than eosinophilic inflammation, by less frequent atopy, by the presence of numerous comorbidities, and by a blunted response to treatment [16]. Due do these differences, whether asthma occurring for the first time in the most advanced ages should be classified as a different disease than asthma that begins early in life and carries over to older ages has recently been questioned [12]. The implication of oxidative stress in adult asthma is well described [17,18]. However, its specific contribution to the geriatric asthma phenotype remains to be established.

This review will focus on the aging-associated alterations in lung immunity and oxidative metabolism, and on their proven or potential contribution to the development of asthma in the elderly. In this perspective, we will also summarize the current knowledge on these aspects in the equine species, as a significant proportion of old horses are affected by a naturally-occurring form of asthma, which is a recognized model for the human disease [19].

## 2. Theories of Aging

Aging is associated with several changes in the physiology of many organelles, organs, and systems. One of the most important is that affecting the immune system. The aged subject presents altered immune responses referred to as immunosenescence. Although it is not a disease by itself [20], aging has been defined as “a generalized, mild, but prolonged type of auto-immune phenomenon” [3]. The process of aging is indeed characterized by a low level of systemic inflammation that is characterized by increased interleukin (IL)-1β, IL-6, and tumor necrosis factor (TNF)-α, and defined as inflamm-aging [5]. Aging cells acquire an irreversible senescence-associated secretory phenotype (SASP), which has beneficial effects such as promoting the clearance of damaged or senescent cells from tissues, but it is taught to facilitate pathogenic pathways implicated in disease development [21]. Chronic cell stress, such as that associated with increased ROS production, activates a pro-inflammatory program, leading to acquisition of the SASP. The implication of oxidative stress as one of the major determinants of disease development in the elderly is well-established [22]. In 1954, Rebeca Gerschman introduced the concept of cellular damage/toxicity that is induced by oxygen free radicals, which until then was considered too reactive to exist in biological systems [23,24]. The free-radical theory or the oxidative stress theory of aging was then proposed by Denham Harman in 1956 [25]. Based on this concept, the physiological formation of ROS during cell metabolism produces oxidative damage to the cell itself, and this, over time, results in a biochemical and physiological decline. It has successively become clear that ROS generation within the mitochondria can alter protein translation as well as causing lipid and DNA damage, eventually leading to a defective function of this organelle in aged subjects. As a consequence, high levels of ROS and mitochondrial dysfunction result in altered or compromised cellular function. This is known as the mitochondrial theory of aging, which is proposed as a correlate of the oxidative stress theory by Denham Harman in 1972 [26], and is now supported by several studies demonstrating an increase in oxidative damage to mitochondrial lipids, proteins, and DNA with age (reviewed by [27]). Given the pro-inflammatory state that is observed in aging subjects and the implication of oxidative damage in cells senescence and aging-associated diseases, a new term has been proposed that underlines the strict relationship that is existing among these processes: oxi-inflamm-aging [10]. Over time, the deficits caused by chronic oxidative stress presumably amplify and contribute to the age-related decline of the physiological organ function.

## 3. Oxidative Stress

**Figure 1:**
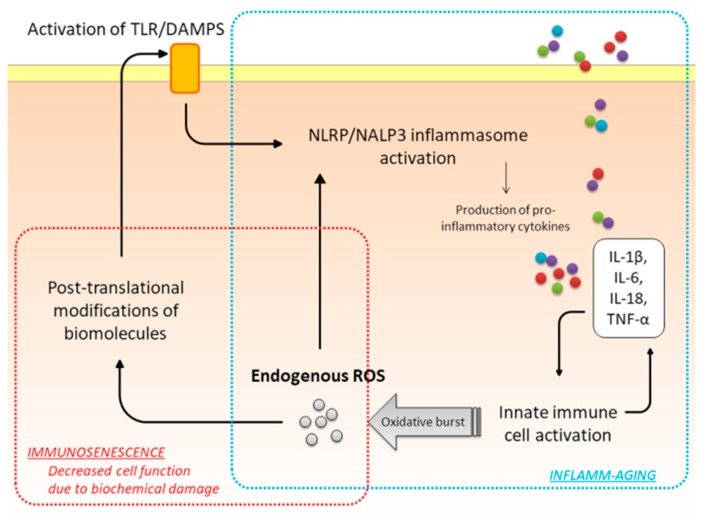
Interrelationship between oxidative stress, immunosenescence and inflamm-aging. Oxidative stress is associated with an increased endogenous reactive oxygen species (ROS) production and/or a decrease in the antioxidant ability within the cell. ROS react with biomolecules (protein, lipids, DNA) leading to cell dysfunction or apoptosis. Byproducts of the oxidative metabolism may activate TLR (Toll-Like Receptors) and DAMPs (Damage Associated Molecular Pattern) receptors, leading to the activation of the innate immune system. In turn, this generates a pro-inflammatory milieu and further increases ROS production, in a positive feedback loop. NLRP-3: Nucleotide-binding domain, Leucine-rich Repeat-containing family Protein 3 (also known as NALP3).

ROS formation is a normal metabolic process that takes place in every cell. ROS are highly reactive molecules with one or more unpaired electron(s) in their outermost shell, such as superoxide (O_2_^•−^), hydroxyl radical (OH^•^), hydroperoxyl radical (HO_2_^•^), nitric oxide (NO^•^), nitrogen dioxide (NO_2_^•^), and peroxyl (ROO^•^). They are by-products of the mitochondrial electron transport of aerobic respiration or of oxidoreductase enzymes. The three major sources of ROS formation within the cells are oxidative metabolism, oxidative burst (or respiratory burst), and exposure to several environmental factors, such as ozone or cigarette smoke [21,22]. Among these, mitochondrial oxidative metabolism is the main contributor to the production of ROS within the cell [28], with ~3% of aerobic cellular oxygen resulting in ROS production [29]. The contribution of oxidative burst to ROS formation becomes important during innate immune system activation. Nicotinamide adenine dinucleotide phosphate (NADPH) oxidases (NOX) are activated in oxidative burst. Whereas, in physiological conditions, about 20% of the oxygen we breathe forms free radicals, their production is much higher in a chronic inflammatory state [30]. Indeed, neutrophils, macrophages, dendritic cells, and monocytes release ROS following activation. The relationship among excessive accumulation of ROS within the cells, immunosenescence, and inflammaging is schematically reported in Figure 1. Mitochondria themselves are a target for ROS-induced damage and for inflammatory mediators. Mitochondria respond to inflammation, allergens, and environmental noxa by altering their transcription/translation machinery, which results in altered function and perpetuates inflammation.

Oxidative stress results from the imbalance between oxidant production and the antioxidant ability of the cell [31]. To counteract the negative effects of excessive ROS levels, cells are provided with a variety of antioxidants, both enzymatic and non-enzymatic. The major enzymic antioxidants are superoxide dismutases, catalase and glutathione peroxidase. Recently, heme oxygenase-1, thioredoxins, peroxiredoxins, and glutaredoxins have also been found to participate in antioxidant defense mechanisms. Vitamins C and E, β-carotene, uric acid, glutathione, albumin, and the tripeptide l-γ-glutamyl-l-cysteinyl-l-glycine are non-enzymatic antioxidants [32]. In oxidative stress conditions, ROS may react with several biomolecules inside the cell inducing ROS-dependent epigenetic modifications and post-translational modifications that negatively affect the cellular function or that trigger immune responses. Post-translationally modified molecules activate Toll-Like receptor (TLR) signaling and NLRP-3 inflammasome, which increases the expression of IL-1, IL-6, TNF-α, and IL-18. These cytokines and chemokines, in turn, activate the innate immune system potentiating ROS production (positive feedback loop). On the other hand, oxidative damage to cellular macromolecules may result in dysfunctional enzymatic activity or in the abnormal accumulation of cellular catabolites, which, respectively, decreases the cell function or induces early apoptosis [8,22]. Oxidative stress is thus strictly linked with immunosenescence and with the SASP.

Besides ROS, also reactive nitrogen species (RNS) play an important role in asthma pathogenesis. Among RNS, nitric oxide (NO) is a byproduct of airway inflammation and tissue damage and a FDA-approved indicator of oxidative stress in the airways [33,34]. NO may also combine with superoxide anions to form peroxinitrite (NOO^−^), which is a very reactive free radical [35]. RNS causes protein nitration and nitrosation, altering their structure and/or function [34].

## 4. Oxidative Stress and Asthma

**Figure 2:**
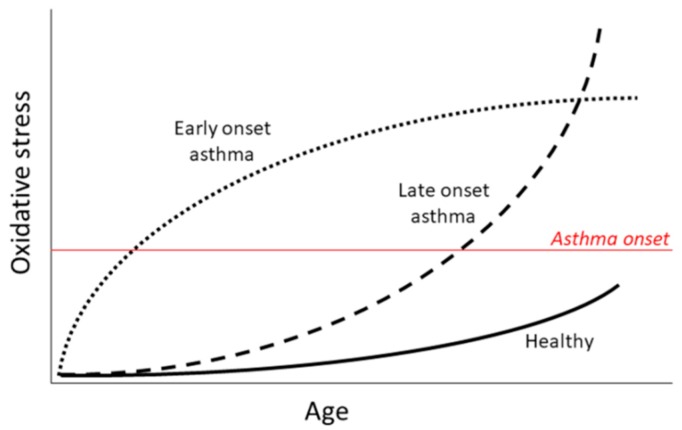
Contribution of oxidative stress in asthma. Oxidative stress has been shown to precede asthma development and to contribute to the disease presentation. We speculate that different patterns of oxidative stress contribute to the early vs. late onset of asthma.

Asthma is an inflammatory disorder of the airways, leading to hyperresponsiveness, obstruction, mucus hyper-production, and airway wall remodeling [36]. Although asthma has long been strictly associated with eosinophilic inflammation and a Th2 biased immune response, more recent data suggest an emerging role for neutrophil involvement in the pathogenesis of the disease and Th17-biased inflammation [37,38,39,40,41,42]. Both eosinophils and neutrophils participate in the innate immune response and produce ROS and RNS in response to different stimuli [6]. There is strong evidence supporting a significant role for oxidative stress in asthma disease and development [17,34,43,44], (Figure 2) which is further increased during asthma exacerbations and in obese subjects [45,46], two conditions that are also associated with increased airway neutrophilia [47,48].

A twofold and fourfold increase was shown, respectively, in the generation of ROS from neutrophils and macrophages in asthmatic as compared with control subjects [49]. Moreover, macrophage ROS generation was associated with an increase in airway responsiveness [50]. In neutrophilic asthma, there is evidence of upregulated nucleotide-binding domain, leucine-rich repeat-containing family protein (NLRP)-3 inflammasome activity [51]. Increased ROS, deregulation of cellular redox status, and mitochondrial stress/damage/dysfunction are considered one of the three key mechanisms that are leading the process of NLRP3 activation [52].

Airway structural cells are important effectors in asthma as they “respond” to the disease-associated inflammatory milieu engendering bronchospasm (smooth muscle cells) and extracellular matrix remodeling/fibrosis (fibroblasts) [36,53], as well as perpetuating inflammation (mainly airway epithelial cells) [54,55]. Both airway smooth muscle cells and fibroblasts secretome is affected by ROS [56,57,58,59,60,61]. Oxidative stress is associated with smooth muscle contraction and proliferation, and induces airway hyper-responsiveness [56,57,58,59,60]. Specifically, NOX4 overexpression has been shown to promote oxidative stress, and consequently airway smooth muscle hypercontractility in asthma [62]. Mitochondria are the main regulators of calcium homeostasis within the cell; their damage/dysfunction induced by oxidative stress results in increased cytosolic levels of calcium, which negatively affects airway smooth muscle contractility [63]. NO increases cell proliferation in cultured human fibroblasts [64], and ROS reactions have been suggested to induce fibrosis either directly or through inflammatory responses [61]. Human lung fibroblasts express MMP-1 when exposed to ROS in vitro [65]. Increased ROS production provokes mucus secretion, cilia damage and epithelial shedding in airway epithelial cells [66,67,68,69], and recent data suggest it can promote the development of an inflammatory environment [70,71]. NOX4 overexpression is associated with bronchial epithelial ciliary dysfunction in human neutrophilic asthma [72]. High levels of hydrogen peroxide are also secreted by bronchial epithelial cells upon histamine activation [73], which might cause a positive feedback loop in asthma. Exposure to aeroallergens increases ROS production and DNA damage in healthy bronchial epithelial cells, blunting their antinflammatory ability via NOX-mediated pathways [54]. High local concentration of NO, along with superoxide anion, has been found to have a cytotoxic effect on airway epithelial cells [74], which may contribute to the epithelial cell shedding that is seen in asthmatic lungs [75]. Damage to the epithelial cell layer can expose underlying sensory nerves to chronic irritants and inflammatory products, resulting in neuropeptide release and the induction of bronchoconstriction [76]. Lastly, the morphological and functional properties of endothelial cells, such as permeability and the expression of adhesion molecules, can be altered by ROS, which also may contribute to the expression of inflammatory mediators [77]. These data indicate that an inappropriate production of free radicals contributes to the destruction of normal tissues and to the prolongation of the inflammatory process [35,65].

Aberrations in oxidant and antioxidant balance can also result in oxidative stress. Asthmatic lungs have reduced SOD and catalase activities in association with a decreased lung function [78,79,80]. Also, there is a suppressed activity of catalase, superoxide dismutase, and glutathione peroxidase in patients with asthma [81]. The antioxidant enzyme CuZnSOD has been found to be decreased in asthmatic airway epithelium and a concomitant increase in superoxide anion production that was was observed in these patients, [82,83]. CuZnSOD is also susceptible to auto-inactivation by hydrogen peroxide [34], indicating that the increased ROS and RNS in asthma not only overcome the pulmonary antioxidant defenses, but act on the latter reducing their function. Recent investigations have demonstrated that MnSOD is down regulated or inactivated (due to its oxidation and nitration) in the airways of asthmatic subjects and its knock down induces epithelial apoptosis [79,83,84]. Airway MnSOD oxidation and nitration also correlate with the severity of asthma [79,84]. A recent study showing that gamma tocopherol-enriched supplement reduces sputum inflammation in asthmatic patients further supports the implication of oxidative stress in clinical manifestations of the disease [85].

## 5. Age-Associated Changes in Pulmonary Structure and (Immune) Function

The aging thorax undergoes structural alterations. The elastic recoil of the lung decreases with aging due to degeneration and redistribution of the collagen and elastic fibers within the tissue, although their quantity remains constant [86]. Collagen becomes stiffer because of increased numbers of intermolecular crosslinks. Elastic recoil reduction could be ascribed to changes in the spatial arrangement and/or crosslinking of the elastic fiber network or to the presence of pseudoelastin (degraded collagen) [87]. A marked dilation of respiratory bronchioles and alveolar ducts is reported with age; the alveolar size also increases in the absence of any destruction of alveolar walls (a paraphysiological condition described as «senile emphysema» [88]), which lessens gas exchange surface (~20% reduction [89]). The increased airspace also lowers the alveolar surface tension, further hindering the elastic recoil of the lungs [90]. Alveolar dilation and extracellular matrix component redistribution decrease airway tethering, increasing airway collapsibility, especially of peripheral conducting airways [91,92]. Finally, chest wall compliance decreases as well in the elderly as a result of the calcification of costochondral junctions, costal cartilage, and degenerative joint disease of the spine, as well as osteoporotic fractures increasing dorsal kyphosis. The age-associated loss of respiratory muscle mass and strength (diaphragm as well as intercostal muscles) also contributes to this phenomenon [92].

The structural changes of the respiratory system with aging results in a physiological decline in lung function in elderly people. The reduced elastic recoil and stiffer chest cause a reduction in the maximum achievable flow in the airways during the breathing cycle. Total lung capacity does not change with aging, however residual volume and functional residual capacity increase (air trapping). As a consequence, vital capacity decreases [87,90]. The closing volume, that is, the lung volume at which peripheral airways start to close during expiration, increases with age. In the elderly, closing volume may equal functional residual capacity (the volume of air present in the lungs at the end of passive expiration). This results in a significant proportion of peripheral airways not contributing to gas exchange during tidal breathing, causing a ventilation/perfusion mismatch, a diminished arterial oxygen tension, and an increase in the alveolar-arterial oxygen gradient. As peripheral airways contribute only marginally to the total airway resistance, these changes are not reflected by significant changes in airway resistance in the elderly [87]. Forced expiratory volume in 1 s (FEV_1_) and forced vital capacity (FVC), decrease with age. However, FVC diminishes later when compared to FEV_1_ and at a slower rate, resulting in an age-associated decline also of the FEV_1_/FVC ratio. As older patients physiologically present an obstructive respiratory pattern, it is critical to use age-adjusted values when interpreting spirometry results in older patients to avoid false positive diagnosis of respiratory dysfunction [14,87,89].

The aged lung not only has a decreased function, but it is characterized by a reduced capacity to respond to environmental stresses and injury. This is due to the effects of cell senescence in both structural and immune cells, and can predispose to disease development. The number of senescent cells in tissues rises substantially during normal aging [93]. Cells can also be induced to senesce via DNA/mitochondrial DNA damage in response to elevated levels of ROS [94]. Mitochondrial dysfunction per se occurs with aging (altered mitochondrial number, structure, motility, and functions) and can contribute to asthma pathogenesis [63]. As mitochondria show cell- and context-specific heterogeneity, the impact of mitochondrial dysfunction on several cell types that are involved in asthma pathogenesis deserves further attention. Structural cell senescence causes a loss or alteration of tissue-repair capacity because of cell cycle arrest in progenitor cells, and because of the SASP-associated production of pro-inflammatory and matrix-degrading molecules. It is overwhelmingly recognized that immunosenescence affects the adaptive response, causing blunted reactions to new antigens. Recent studies have also identified that immunosenescence occurs at the innate system level [4], causing a state of chronic subclinical inflammation of the organism (immune-aging). Among the forty cell types that are found within the lungs [95], neutrophils, macrophages, natural killer cells, dendritic cells, as well as airway epithelial cells contribute the most to the innate immune response. Pulmonary levels of complement proteins also have been found to increase with age [48]. Neutrophils and macrophages are first line of defense against pathogens. Airway but not systemic neutrophils are increased in healthy patients that = older than 50 years [96,97,98,99,100], indicating that sputum neutrophilia can be dissociated from airway symptoms and could create a favorable background for the development of age-related lung diseases [96]. The physiological airway neutrophilia in the elderly may result from increased chemoattractant concentration or exaggerated chemotactic response, delayed apoptosis, decreased clearance ability by macrophages, or by an altered expression of neutrophil or vascular adhesion molecules. A recent study has shown a positive correlation between age and sputum neutrophilia, as well as IL-8 and TNF-α levels in the exhaled breathing condensate of healthy individuals [98]. With increasing age, however, neutrophil chemotaxis to the site of inflammation is reduced [6]. While there are no reports in the literature showing aging-associated delay in apoptosis, several studies show that apoptosis is increased in circulating neutrophils in the elderly [101,102,103]. Aged macrophages also have reduced phagocytic capabilities [104,105,106]. Adhesion is reported to be unchanged or slightly increased, thus reducing chemotaxis. The increases in the expression of neutrophil adhesion molecules CD11b and CD15 in aged subjects [107,108] are likely to enhance neutrophil adhesion to endothelial cells and contribute to the impaired chemotaxis, as observed in neutrophils of aged patients. Besides the increased number of lung neutrophils, their function also is partly impaired in aging. Basal ROS and NO production are increased in neutrophils from aged donors [108]. Conflicting data exist concerning the ability of neutrophils from aged patients to produce ROS in response to diverse external stimuli. Overall, ROS production is maintained, but specific signaling pathways to evoke ROS production may be blunted in the elderly [13,109,110,111,112], which may predispose to external insults. Neutrophil phagocytic activity decreases with aging [113]. Lipopolysaccharide (LPS)- and interleukin-8 (IL-8)-induced neutrophil extracellular trap (NET) formation also exhibits a significant age-related decline [109]. Elderly subjects experience more frequent and more severe respiratory infections compared to younger people, which is likely due to the reduced microbicidal activity and immune response of pulmonary neutrophils [6]. The airway microbiome is also likely to be influenced by immunosenescence and inflamm-aging in the elderly, as specific phenotypes of asthma have been linked with different microbiota [114]. No information, however, is currently accessible addressing the microbiome of elderly asthmatics (>65 years), and a definitive causative link between age-associate changes in the microbiota and late-onset asthma has not been established [115,116].

Macrophages are present both within the alveoli and within lung parenchyma. Alveolar macrophages act as the first immune defense of the lung by clearing airborne and microbial particles that reach the alveoli during breathing. In response to various signals, macrophages may undergo classical M1 activation (stimulated by TLR ligands and IFN-γ and characterized by pro-inflammatory activity and ROS production), or alternative M2 activation (stimulated by IL-4/IL-13 and considered as anti-inflammatory but also associated with the development of fibrosis or allergies) [117]. Macrophage number is not affected by aging [118], while the ability of these cells to polarize into M1 or M2 seems to be blunted in the elderly [119]. The phagocytic capacity of alveolar and pulmonary macrophages declines with age [104], which could also contribute to the increased susceptibility to respiratory infections in the elderly and possibly to the subclinical airway neutrophilia that is observed in this age group [96,97,98,99,100]. Studies investigating the effect of age on the ability of human alveolar macrophages to produce ROS are lacking. Animal studies suggest that macrophages from old animals produce higher ROS levels or have an impaired antioxidant shield [120]. A reduced number of monocytes that were harvested from old subjects generated altered levels of IL-6 and TNF-α upon TLR1/2 stimulation when compared to the young group [121]. Also, human macrophages that are derived from in vitro differentiation of monocytes had decreased the expression of TLR3 mRNA and intracellular protein in older subjects [122], and TLR-induced expression of B7, a protein that is involved in antigen presenting cell-T cell cross-talk, is decreased in the elderly [123]. An altered expression of MHC class II molecules has been shown in this age group [124], which could contribute to a poor T cell response. Collectively, these data suggest that the elderly are characterized by a blunted inflammatory response that may also dysregulate the adaptive immune system through altered molecular cross-talk.

Natural killer cells are effectors of innate immunity that are playing important functions during the response to viral infections and in self-tolerance, and their number is increased in the elderly [6,13]. However, this is not associated with enhanced global cytotoxicity and may constitute a compensation mechanism for the reduced cytotoxicity of the single cell [113]. Both function and phenotype of natural killer cells are altered with advanced age [125,126,127], which impairs their response to viral infections [128].

The airway and alveolar epithelial cells represent the first barrier against external noxa in the lungs, and have important immunomodulatory functions. The airway epithelium is mostly composed by ciliated cells and mucus producing cells. The effects of aging on these cells in man have been poorly investigated; few studies have shown that mucociliary clearance decreases in healthy elderly [129,130]. These results are consistent with the reduced ciliary beat frequency that was observed in aged versus young mice [131]. Old mice also have a reduced number of epithelial cells due to increased apoptosis, and resulting in epithelial thinning [132]. Virus-induced damage of club cells and their subsequent regeneration was similar in young and aged mice, however [133]. An age-related reduction in the induction of Nrf2-regulated anti-oxidant genes has been reported in human bronchial epithelial cells [134].

The alveolar epithelium is made by alveolar type I and type II epithelial cells (pneumocytes). Type I pneumocytes are squamous cells that cover most of the alveolar surface (>90%). They have barrier functions and are involved in the process of gas exchange between the alveoli and the pulmonary capillary network. Type II alveolar cells are relatively numerous (60% of pneumocytes), despite the fact that they cover only <5% of the alveolar surface; their main function is the secretion of pulmonary surfactant. Contrarily to type I pneumocytes, type II cells do proliferate and, following differentiation, serve as progenitor for type I pneumocytes. Damage to type I and II pneumocytes is exacerbated, and regeneration of AT2s and their precursors is significantly delayed, in aged mice [133]. In this species, alveolar stem cell exhaustion, caused by a reduced ability of type II pneumocytes to proliferate (cell senescence), induces a pulmonary pro-inflammatory response that is characterized by augmented bronchoalveolar and parenchymal mononuclear inflammation, and by an increased susceptibility to injury [135]. This finding supports cell senescence as a potential trigger of chronic pulmonary diseases. Surfactant is stored in specialized vesicles in the cytoplasm of type II pneumocytes, called lamellar bodies. The levels of surfactant protein-A (SP-A) and SP-D, two members of the collectin family that play important and unique roles in pulmonary defense against inflammation and oxidative stress [136,137], increase during aging both in the alveolar lining fluid and in plasma [138,139]. Aging-associated changes have also been reported in surfactant composition. Pro-inflammatory cytokines and the activity of myeloid peroxidase increase with age in the alveolar lining fluid [138].

Dendritic cells lie at the interface between the innate and adaptive immune responses. Dendritic cell function is impaired with age. Specifically, dendritic cells from aged subjects display decreased phagocytic and migratory capacities [140,141,142]. However, they are characterized by an increased basal activation of nuclear factor kβ and the secretion of several pro-inflammatory molecules, such as IL-6, TNF-α, and metalloproteinases, which are able to activate bronchial epithelial cells [143,144] and potentially contribute to the increased reactivity to self-antigens, such as intracellular DNA that is observed in aged subjects [144]. This priming of dendritic cells induces an enhanced T cell proliferation and may contribute to inflamm-aging [144]. TLR function of dendritic cells from healthy subjects ≥65 years of age is decreased when compared to that of subjects 21–30 year old [145]. A decline in the antigen presenting efficacy of dendritic cells harvested from aged subjects has also been observed [142], which negatively affects immune acquired response.

Both T and B cells are decreased in number in aged subjects, which is mainly due to a reduction of naïve T and B cells [146,147,148]. T cells from aged subjects show a reduced activation with decreased proliferation and blunted response to external antigens [149]. Among T cells, the CD8^+^ cell subset seem to be affected to greater extent [150,151,152], suggesting that CD4^+^ cells are subject to stricter homeostatic mechanisms. A shift in T-helper cell subpopulations has been proposed with advanced age. Th17 cells are predominantly observed in aged subjects with asthma [153,154,155], as opposite to the Th2 inflammatory milieu that is observed in most young asthmatic patients [38]. As Th17 cells could develop from the same lineage as the anti-inflammatory regulatory T-cells (Tregs) [156], it is plausible that a preferential shift toward Th17 response may decrease Tregs and facilitate the development of a pro-inflammatory milieu in the elderly. B cells show an impaired capacity for response to new antigens during aging, a blunted response to antigens previously encountered due to a reduced clonal expansion capability of memory B cells and lower level of circulating antibodies. Finally, the antibodies that are produced by B cells from aged subjects are characterized by lower affinities with antigens and decreased opsonizing abilities [113].

In summary, the aging lung is a relatively oxidized environment [138], which can cause peroxidation of membrane lipids, depletion of nicotinamide nucleotides, rises in intracellular calcium ions, cytoskeleton disruption, and DNA damage [22]. The main cellular sources of reactive oxygen species in the lung include neutrophils, eosinophils, alveolar macrophages, alveolar epithelial cells, bronchial epithelial cells, and endothelial cells [157,158,159]. Although the aging-associated alterations of these cell types in asthmatic patients have been investigated in a limited number of studies, evidence exists linking such changes with the onset of asthma in the elderly [14,160].

## 6. Asthma in the Elderly: A Different Disease? [12]

The current prevalence of asthma is reported to be from 4% to 13% of adults >65 years [161]. Overall asthma prevalence in the elderly decreases with advancement of age, while the proportion of affected women increases with age [162]. However, this number is likely an underestimation, as asthma is frequently underdiagnosed in this age group both because the frequent presence of respiratory comorbidities and the physiological decline in lung function with aging [16,163]. For the same reasons, older asthmatics show a more severe disease phenotype when compared to younger patients. Therefore, asthma-associated morbidity and mortality increase with age. Elderly patients with asthma are at the greatest risk for frequent hospitalizations and they also are >5 times more likely to die from their disease compared to younger individuals [161], possibly also because of the age-related impairment in perception of breathlessness. It is recognized that lung epithelial cell senescence may contribute to the pathogenesis of late-onset asthma or to persistence of asthma into later years [13]. Due to the age-associated changes in lung (as well as other organ) function and immunity that have a repercussion on the clinical presentation and management of asthma in the elderly, a different approach to the disease is warranted in this age-group [12]. Older age often represents an exclusion criterion for eligibility in prospective studies and clinical trials, which is why the pathophysiology and treatment of asthma in older patients are not as well characterized as it is in younger adults and children.

The characterization of the geriatric asthma phenotype represents a significant knowledge gap in the asthma literature. The high morbidity and mortality that is recognized in elderly patients with asthma, together with the overall aging process of the global population and its healthcare associated costs, has prompted physicians to pay greater attention to this asthma phenotype. An in-depth process of classification of the different asthma phenotypes based on lung function and clinical outcomes in elderly patients has recently been implemented [164]. Elderly patients with asthma can be divided in two groups: the early-onset phenotype (or long-standing asthma), and the late-onset phenotype [14]. Early-onset elderly asthmatics have developed the disease in the first two decades of life, are characterized by a Th2 biased immune response, and by the development of a severe partly-irreversible obstruction. In these patients, oxi-immune-aging effects are likely to sum up with asthma-related characters determining disease expression in the adulthood/old age. On the other hand, late-onset elderly asthmatics are characterized by mixed Th2/Th17 eosinophilic-neutrophilic inflammatory response, by the occurrence of severe exacerbations, and are less atopic [14,38]. While there is no doubt that oxi-immune-aging is involved in disease pathobiology also in these patients, how it contributes to the pathogenesis (occurrence) of the disease in this cohort remains to be established.

Elderly asthmatics are more prone to show a predominantly neutrophilic inflammation [165,166,167]. Accumulating evidence suggests that oxidative stress plays an important role in neutrophilic asthma. NOX activity is regulated by Th2-type cytokines, such as IL-4 and IL-13, important mediators of asthma, and stimulate the production of IL-8, which is a potent neutrophil chemoattractant. A mouse model of asthma lacking two members of the NOX family was characterized by reduced expression of Th2 cytokines in bronchoalveolar fluid, by a reduced neutrophil influx to the airways and by reduced IL-6 expression [168]. IL-6 promotes granulopoiesis in the bone marrow [169]. Moreover, stimulating peripheral blood neutrophils from human asthmatics with IL-6 induces an overexpression of IL-17A and IL-17F, which may establish a positive feedback loop for neutrophil recruitment. Recent studies have reported increased oxidative damage and NOX4 expression in human neutrophilic asthma in association with a dysfunctional bronchial epithelial ciliary apparatus [72]. Studies on neutrophilic asthma are still limited, although they have been growing in number in the last decades. Moreover, whether the implications of bronchial neutrophilia are the same in young and old asthmatic patients has not been studied. The age-associated decline in mitochondrial function and immunosenescence suggest that it may not be the case. For this reason, data obtained in studies that were performed on young/adult neutrophilic asthmatics should be transposed with caution in the context of geriatric asthma. It is possible the increased airspace neutrophils contribute to greater severity of asthma in the elderly both directly, by maintaining a pro-inflammatory milieu within the airways, and indirectly, by fostering airway remodeling [13,165]. By-products of activated neutrophils, such as metalloproteinases and elastases, significantly alter the structure and/or composition of the airway scaffold, the extracellular matrix. The existing literature indicates that elderly asthmatics present greater airway remodeling when compared to younger patients in terms of airway wall thickness/area [170,171], although disease duration might be a source of bias in these types of studies [170].

## 7. Severe Equine Asthma: A Model of Oxi-Inflamm-Aging

**Figure 3:**
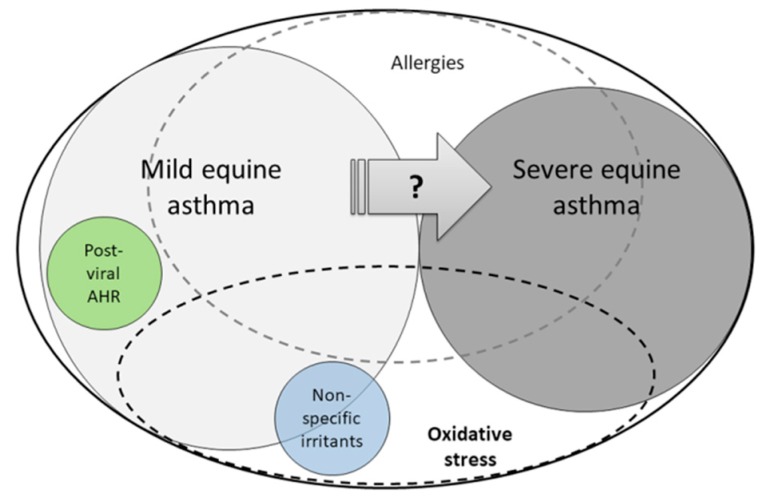
Equine asthma phenotypes. Oxidative stress plays a role in both mild and severe forms of equine asthma.

Severe equine asthma, also known as heaves, RAO (recurrent airway obstruction), or SPAOPD (summer-pasture associated obstructive pulmonary disease) is a spontaneously occurring disease of horses and a recognized model for human asthma [19,172,173]. Among the equine asthma phenotypes (Figure 3), the severe form describes horses that are experiencing episodes of dyspnea at rest triggered by hay dust antigens and reversible with the administration of bronchodilators, in the absence of infections. Other non-specific clinical signs that may accompany the disease are chronic cough and nasal discharge. Severe equine asthma is characterized by airway neutrophilia (defined as >20–25% neutrophils in bronchoalveolar lavage fluid cytology [174]), airway remodeling, and bronchospasm.

Severe equine asthma is a disorder whose occurrence is determined by the interplay of genetics and environmental factors, and that manifests clinically only in adult and geriatric horses. Whether its subclinical development starts early/earlier in the horse’s life has still to be established. Different hypotheses have been proposed based on which severe equine asthma is the end-stage disease that follows mild equine asthma or the sequela of respiratory infections occurring in predisposed animals [174,175]. What has been shown is that even occasional respiratory insults (as those commonly observed during mild asthma episodes or during viral respiratory infections) increase the risk of severe equine asthma occurrence 7 to 10 folds in horses [176]. Nevertheless, a definitive causative relationship has not been established for either of these factors. A study has shown how aging also represents a risk factor for the development of severe equine asthma in horses. While animals that are older than five years are already five times more at risk of developing asthma as compared to younger animals, in horses aged >15 years the risk increases up to 18 times more [177]. The reasons that are related to this finding have not been elucidated however.

Immunosenescence occurs in horses and affects both the innate and the acquired immune system [178,179]. Evidence also exists supporting the occurrence of inflamm-aging in this species [180,181,182]. Severe equine asthma is a chronic inflammatory condition associated with a dysregulated innate and acquired response mirrored, respectively, by neutrophilia/chronic systemic inflammation [183,184] and by an overexpression of Th1, Th2, and/or Th17-type molecules [185,186,187,188,189]. The discrepancies in Th1, Th2, and Th17 related cytokines thata are observed in different studies could be related to geographical/environmental factors, methodological factors, or to the presence of several endotypes of the disease that is characterized by different molecular pathways. Further work on large samples will have to support the latter hypothesis. As severe equine asthma mainly affects adult and geriatric horses, the contribution of immunosenescence, inflamm-aging, and age-related oxidative stress to its development should not been underestimated.

A recent study has showed that adhesion, oxidative burst, and phagocytosis were all found to be similar in peripheral blood neutrophils from healthy aged and adult horses, whereas leukotriene B4-induced chemotaxis was increased in older animals (with only one old horse behaving differently) [190]. The significance of these findings on asthma susceptibility in the aged horse at this time remains unclear however. Asthmatic horses present increased levels of circulating low-density neutrophil-like granulocytes that show an increased response to stimulation in vitro in terms of neutrophil extracellular trap formation. Whether this is associated with, or mediated by, oxidative mediators has not been investigated however [191]. Few studies have investigated whether age influences bronchoalveolar lavage fluid (BALF) cytological profiles in horses, with no significant results [182,192] or with only a decrease in lymphocyte detected [193]. A reduced concentrations of surfactant phospholipids in aged compared to younger horses has been shown, however [192]. Diminished levels of surfactant phospholipids were also observed in severely asthmatic horses in remission of the disease compared to controls, with a further decrease occurring during periods of exacerbation that was possibly due to the airway neutrophilia [194,195]. Of note, a mechanism that accounts for the alteration in surfactant content and bioactivity is ROS-induced lipid peroxidation [196]. Age-related decreases in partial pressure of arterial oxygen and carbon dioxide, as well as an increase in alveolar to arterial pressure gradient, have been reported in horses >20 years of age when compared with 3–8-year-old horses [197]. These changes likely are the result of decreased diffusion capacity and increased ventilation perfusion imbalance in the lungs of older horses. Whether «senile emphysema» occurs in older horses is not known to date. However, severe equine asthma was initially named «chronic alveolar emphysema», and early reports exist showing a dilation of the alveoli in this disorder [175]. Whether this could have been misinterpreted as emphysema (loss of alveolar tissue) remains to be established.

Old horses have reduced levels of circulating CD4^+^, CD8^+^, Tregs, and B cells, as well as a blunted proliferative response of lymphocytes whose mechanism has not been established. When activated, however, peripheral blood mononuclear cells from old horses produce greater levels of TNF-α and IFN-γ as compared to younger animals, suggesting that a switch from an anti-inflammatory to a pro-inflammatory action of these cells occurs with age [178,179,198,199,200]. The proportion of IFN-γ-producing lymphocytes also increases with age in BALF [182], which *per se* could represent a favorable environment preventing the development of asthma, as it would shift the Th-type response towards type 1 immunity instead of type 2 [178]. Recent studies have shown that oxidative endogenous DNA damage is increased in the peripheral blood mononuclear cells of old when compared to adult healthy horses [201] and in asthmatic horses in clinical remission of the disease as compared to age-matched healthy controls [202]. A correlation between endogenous DNA damage and glutathione concentration was also observed in healthy animals, suggesting that antioxidant defenses are not overwhelmed in the aged horse [202]. This finding is supported by another study in which blood lipid peroxidation and antioxidant levels were similar in old and in mature, but younger, horses [203].

Data on the amount and on the causes/consequences of oxidative stress in the pulmonary environment is growing in the last years, and accumulating evidence supports a role for this mechanism in equine asthma [204]. Asthmatic horses in remission have lower concentrations of ascorbic acid in their BALF when compared with healthy horses [205], possibly increasing their susceptibility to oxidative damage during exacerbation-induced inflammation. The 8-epi-PGF2alpha, a marker of oxidative stress, is increased in lungs of asthmatic horses, and, when administered by inhalation, induces a mild but significant bronchoconstriction [206]. Antioxidant levels increase during disease exacerbations [207,208], suggesting a maintained ability to cope with increased oxidative stress. Despite BALF neutrophilia correlating with hydrogen peroxide in exhaled breath condensate in asthmatic horses [209], acute neutrophilic airway inflammation does not produce oxidative stress in these animals indeed [210]. BALF neutrophilia and exhaled hydrogen peroxide negatively correlate with the plasmatic levels of ascorbic acid, an important antioxidant in the horse [205]. Decreases in BALF levels of ascorbic acid after antigen exposure correlate with an increased respiratory resistance in asthmatic horses [210]. Finally, antioxidant supplementation resulted in improved oxidative balance but did not affect BALF composition or clinical presentation of the disease in two studies [211,212]. In a recent study, the administration of omega-3 fatty acid supplementation improved lung function parameters in asthmatic horses in absence of a reduction of the oxidation marker 8-epi-PGF2alpha [213]. Training also has been reported to improve the antioxidant ability of adult horses [214].

## 8. Unanswered Questions in Geriatric Asthma and Possible Contribution of the Equine Asthma Model to Knowledge Advancements

Recently, a new concept, called geroscience, was proposed to understand the putative role of aging in the appearance and development of age-related diseases [4]. The oxidative metabolism has an important role in aging and in the regulation of immune responses, and oxidative stress may be involved in the development of age-related diseases. Oxidative stress has been recognized to play a major role in determining and maintaining the low-grade inflammation that was observed in aging (inflamm-aging) [22]. Age-related changes in the respiratory system and oxidative metabolism can coincide with asthma and may contribute to the disease expression in the elderly. Further research is needed to elucidate redox mechanisms that pertain to the progression of asthma in the elderly as certain altered immune responses could facilitate the pathogenesis of this phenotype of the disease. Given the importance that mitochondrial dysfunction can have in asthma pathogenesis, efforts should be directed also towards characterizing mitochondrial specific alterations in different cell types and their contribution to disease development or progression. A recent review has highlighted the need to identify appropriate in vitro and in vivo models to explore mitochondrial dysfunction in the airways [63].

Moreover, current asthma treatments may be less effective in the elderly population [215]. Older patients are less responsive to emergency medication [216], although poor inhaling technique, airway remodeling, concurrent medication, and comorbid conditions may all contribute to this effect [215]. Data on airway remodeling in the elderly patients are scarce, as well as on the effects of asthma-treatment as the presence of comorbidities or other treatments—frequent in old patients—are common exclusion criteria in large clinical trials [217]. Geriatric-specific guidelines are not available for the diagnosis and treatment of asthma and future research in this field is of paramount importance in the perspective of personalized medicine.

In this panorama, severe equine asthma represents a suitable model for studying the contribution of oxi-inflamm-aging to asthma development and presentation in the elderly, and its possible implication in treatment response. The main strengths of this model are the natural occurrence of the disease and the long lifespan of the animals. Horses are a long-lived species when compared with other asthma models (i.e., rodents) with a mean lifespan of 25 years, estimated to be equivalent to 71 year of age in people, which can extend up to 40 years in some subjects [179]. On the other hand, these features of the disease could represent drawbacks if we aim at studying the immunity mechanisms that are leading to the occurrence of the disease, as currently, there are no means to predict animals that will eventually develop the disease. Efforts should be directed toward a thorough characterization of immunity dysfunctions or inflammatory pathways/endotypes in mild equine asthma, and their relationship with the outcome of the disease (possible evolution toward severe equine asthma or complete disease remission).
